# The Relationship between Body Composition and Muscle Tone in Children with Cerebral Palsy: A Case-Control Study

**DOI:** 10.3390/nu12030864

**Published:** 2020-03-24

**Authors:** Paweł Więch, Agnieszka Ćwirlej-Sozańska, Agnieszka Wiśniowska-Szurlej, Justyna Kilian, Ewa Lenart-Domka, Agnieszka Bejer, Elżbieta Domka-Jopek, Bernard Sozański, Bartosz Korczowski

**Affiliations:** 1 Institute of Health Sciences, College of Medical Sciences of the University of Rzeszow, University of Rzeszow, 35–959 Rzeszow, Poland; sozanska@ur.edu.pl (A.Ć.-S.); wisniowska@vp.pl (A.W.-S.); justynakilian110@gmail.com (J.K.); e.domka@op.pl (E.L.-D.); agnbej@wp.pl (A.B.); elasia05@poczta.onet.pl (E.D.-J.); benieks@poczta.onet.pl (B.S.); 2 Institute of Medical Sciences, College of Medical Sciences of the University of Rzeszow, University of Rzeszow, 35–959 Rzeszow, Poland; korczowski@op.pl; 3 Clinical Regional Rehabilitation and Education Centre for Children and Adolescents in Rzeszow, 35-301 Rzeszow, Poland

**Keywords:** electric bioimpedance, Ashworth Scale, nutritional assessment, rehabilitation

## Abstract

The monitoring of children with cerebral palsy (CP) should include a precise assessment of the nutritional status to identify children and adolescents at risk of nutrition disorders. Available studies assessing the nutritional status of children with CP mainly focus on the relationship between body composition and the coexistence of motor dysfunctions, frequently overlooking the role of muscle tone. Therefore, the aim of this study was to assess the relationship between body composition and muscle tone in children with CP. In a case-control study (*n* = 118; mean age 11 y; SD = 3.8), the children with CP presented various stages of functional capacities, corresponding to all the levels in gross motor function classification system (GMFSC), and muscle tone described by all the grades in Ashworth scale. The control group consisted of healthy children and adolescents, strictly matched for gender and age in a 1:1 case-control manner. The children with CP were found with significantly lower mean values of fat-free mass (FFM kg = 29.2 vs. 34.5, *p* < 0.001), muscle mass (MM kg = 18.6 vs. 22.6, *p* < 0.001), body cell mass (BCM kg = 15.1 vs. 18.3, *p* < 0.001), and total body water (TBW L = 23.0 vs. 26.7, *p* < 0.001). The same differences in body composition were identified with respect to gender (*p* < 0.01 respectively). Moreover, children with higher muscle tone (higher score in Ashworth scale) were found with significantly lower values of fat mass (FM), FFM, MM, BCM, and TBW (*p* < 0.05). The findings showed lower parameters of body composition in the children with CP compared to the healthy children, and a decrease in the parameters coinciding with higher muscle tone in the study group. This observation suggests that it is necessary to measure muscle tone while assessing nutritional status of children with CP.

## 1. Introduction

Cerebral palsy (CP) is considered a non-progressive dysfunction of the central nervous system that is largely associated with motor control centers [1]. This injury often involves the descending pathways of the central nervous system, causing inappropriate muscle activation and hypertonicity as a consequence [2]. The three basic types of CP include spastic, dyskinetic, and atactic forms, however, each of these forms is characterized by motor and posture disorders [3]. Although the primary damage to the central nervous system is static, secondary musculoskeletal complications develop over time, including soft tissue contracture [4]. It is believed that hypertonicity causes increased energy consumption during movement, and muscle contractures negatively affect the gait pattern [5]. These changes directly affect general health, mobility, and independence in everyday life, increasing the level of disability in children with CP [6,7].

Studies show that increased muscle tone also significantly increases difficulty in food intake and swallowing, and the consequent nutritional deficiencies further exacerbate disorders in the development of children with CP [8]. Possible effects of malnutrition in children with CP include poorer respiratory function, immune and circulatory dysfunctions, and an increased risk of irreversible systemic metabolic disorders [9,10].

Studies revealed that, in many cases, energy consumption in children with CP is insufficient to meet their energy needs compared to healthy children [11]. Therefore, body composition analysis and estimation of their caloric demand should be an important action undertaken in children with CP [12,13]. Despite the common belief that it is necessary to systematically evaluate nutritional status in children with CP, such evaluation is extremely difficult due to many factors [9]. The co-existing contractures and pareses frequently make it impossible for these children to assume a standing position. Furthermore, because of their growth retardation it is difficult to apply the available centile grids enabling assessment of weight and growth rates, and commonly used in evaluating nutritional status of normally developing children [14]. Moreover, the interpretation of body fat using % fat is also affected by low fat-free mass given the interdependency of fat and fat-free mass to estimate % fat [15]. In view of these significant limitations linked with other measurement methods, bioelectrical impedance analysis (BIA) is more and more commonly applied in assessing nutritional status recognised by clinicians monitoring nutritional status of children with CP [12,13]. 

García Íñiguez et al. showed that there is a direct correlation between resting energy expenditure, total energy expenditure and fat mass, muscle mass, and total body water in children with CP [16]. Many studies to date have also shown a relationship between energy demand and muscle tone in children with CP [2,17,18,19]. These reports suggest a relationship between muscle tone and body composition and energy requirements in children with CP. In addition, as indicated by Lindén et al., the level of muscle tone is subject to the greatest changes in children with CP during the first years of life, while demonstrating a relationship with growing disability in the gross motor skills (GMFCS) [20].

A review of the literature shows that, despite a wide range of studies on body composition in children with CP, no study has yet been conducted directly assessing the relationship between muscle tone in the Ashworth scale and specific body composition parameters. Therefore, the authors decided to assess the relationship between muscle tone as measured with the Ashworth scale and body composition of children with CP.

## 2. Materials and Methods

### 2.1. Ethics

The study was approved by the institutional Bioethics Committee at the University of Rzeszow (Resolution No. 2017/12/12) and by all appropriate administrative bodies. The study was conducted in accordance with ethical standards laid down in the relevant version of the Declaration of Helsinki (64th WMA General Assembly, Fortaleza, Brazil, October 2013) and in Polish legal regulations. The study was conducted according to the STROBE criteria and registered with www.researchregistry.com (IUN research registry researchregistry4673).

### 2.2. Subjects

The study conducted between June 2018 and July 2019 involved a group of 118 children and adolescents (76 boys, 64.4%) with CP receiving inpatient treatment at the Neurological Rehabilitation Ward at the Clinical Regional Rehabilitation and Education Centre for Children and Adolescents in Rzeszow. The mean age of the children was 11 years (SD: 3.8). The inclusion criteria were as follows: medical diagnosis of CP, age 4–18 years, functional performance corresponding to all the levels in the GMFCS, no epileptic seizures in the six months preceding the examination as well as informed written consent, signed by parents or legal guardians, and by the adolescents over 16 years of age. The children were excluded from the study if their medical history contained neurological disorders other than CP, acute inflammatory processes, or an epileptic seizure during the previous six months. Information on the type of CP, identified based on World Health Organization International Classification of Diseases and Related Health Problems ICD-10 [21], was obtained through a review of the patients’ medical documents.

The functional level of participants (study group) was classified according to GMFCS [22,23]; 56 children with CP were in Level II (47.5%), 32 were in Level I (27.1%), 17 were in Level IV (14.4%), 9 were in Level V (7.6%) and 4 were in Level III (3.4%).

Majority of the children with CP presented the spastic type of the condition (spastic, *n* = 94, 79.7%). The study group also included children with mixed CP (*n* = 16, 13.6%), ataxic CP (*n* = 6, 5.1%) and unclassified types of CP (*n* = 2, 1.7%). Muscle tone, assessed using Ashworth scale [24,25], in most children was at Level 1 (*n* = 54, 45.8%). Further, 35 children (29.7%) presented Level 2, 14 children (11.9%) presented Level 3, 13 children (11.0%) presented Level 0, and 2 children (1.7%) presented Level 4.

The control group consisted of the same number of children and adolescents attending primary, middle and secondary schools in urban and rural areas. Participation in the study was voluntary and anonymous. The purposes and procedures of the study were explained, and informed consent was obtained. The personal data of subjects were protected by assigning each participant with a code in the form of a digital number. The healthy participants and those with CP were strictly matched for age (the nearest birth date) and gender in a 1:1 case-control manner. The precise characteristics of both groups are described in supplementary materials (Table S1).

### 2.3. Assessments

Detailed medical history of the children with CP, including the diagnosis, the course and treatment of the disease, as well as the comorbidities, was retrieved from the patient records. These assessments were supplemented with information on the children’s eating habits, based on a questionnaire evaluating the typical intake of basic product groups per week.

The level of spastic muscle tone in children with CP was assessed in the Clinical Regional Rehabilitation and Education Centre for Children and Adolescents in Rzeszow with original Ashworth scale, covering five levels: 0 (no increase in tone), 1 (slight increase in tone giving a catch when the limb is moved in flexion and extension), 2 (more marked increase in tone, but limb is easily flexed), 3 (considerable increases in tone, passive movement difficult), and 4 (rigid limb) [24,25].

The level of functional mobility was assessed using GMFCS [26,27]. GMFCS is an objective classification method, which consists of five levels: I (children with minimal or no disability in the field of mobility in the community), II (children are able to go inside and outside with restrictions, but do not use devices), III (children are able to go inside and outside with restrictions and use various aids), IV (children use methods of mobility that require physical assistance or powered mobility in most settings), V (children totally dependent on assistance in mobility). All the children were assessed for height and weight. The measurements were performed under standard conditions, in an upright position, barefoot, and in a fasting state. Body weight and height were assessed with an accuracy of 0.1 kg/0.1 cm using a digital scale (Radwag 100/200 OW, Radom, Poland). Body mass index (BMI) was calculated as weight (kg)/height (m^2^) [kg/m^2^].

The bioimpedance parameters: R (Ω) - resistance (is the opposition offered by the body to the flow of an alternating electrical current, and it is inversely related to the water and electrolyte content of tissue) and Xc (Ω)- reactance (is related to the capacitance properties of the cell membrane, and variations can occur depending on its integrity, function, and composition) were obtained using bioelectrical impedance analyser AKERN BIA 101 Anniversary (Akern SRL, Pontassieve, Florence, Italy). The results were analysed using dedicated software (Bodygram1_31 from AKERN, Pontassieve, Florence, Italy). The equations used by the software to assess the specific parameters are restricted property of the company, but to a significant degree, they are based on computed algorithms developed by Sun S. et al. [28]. The measurements were performed between 7:00 a.m. and noon, on an empty stomach, in the supine position, with abducted upper (30°) and lower (45°) limbs, following at least a 5-min rest. A tetrapolar electrode arrangement was applied with contralateral recording mode. The amplitude of the measured current was 800 μA, sinusoidal, 50 kHz. To ensure that the results were reliable and reproducible, two measurements were performed, one after another. Disposable electrodes were placed on the dorsal surface of the right arm (above the wrist) and the right leg (on the ankle). All the measurements were performed according to guidelines described by other authors [29,30,31,32].

The following measures were analysed: fat mass (FM) (kg and %), fat free mass (FFM) (kg and %), total body water (TBW) (L and %), muscle mass (MM) (kg and %), body cell mass (BCM) (kg and %), as well as body cell mass index (BCMI). Additionally, phase angle (PA) was calculated, based on resistance and reactance (PA = arctan (Xc/R) × (180/π), where arctan denotes arctangent, Xc is reactance, R, resistance, and π is 3.14) [33].

### 2.4. Statistical Analysis

Data are reported as mean and 95% confidence interval (CI) for quantitative measures and as percentages for all categorical variables. The independent samples t-test was used to compare the mean level of somatic features and body composition in the group of children with CP and in the control group. In order to monitor age during statistical calculations, the ANCOVA model, which connects ANOVA with regression was used in the examined groups (size effect, the value of F-statistic together with the numbers of degrees of freedom and *p*-value were given). ANCOVA removes the impact of one or more undesirable variable from dependent variable. In this paper, ANCOVA models were used to assess the significance of differences in the mean level of body composition parameters between the groups of children with different levels of disease advancement with respect to the age of children. The correctness of the inference made using ANCOVA models was verified by analyzing the residuals distribution, both in terms of their normality and the occurrence of outliers. Calculations were performed with Statistica 12.5 (StatSoft, Inc., Tulsa, OK, USA). A *p*-value below 0.05 was considered statistically significant.

## 3. Results

Table 1 shows the characteristics of the groups. All analyzed parameters significantly differentiated children from CP and control group (boys). There were no significant differences in BMI values and tissue resistance parameters (R and Xc) in girls.

Body composition parameters were significantly different between the CP and control groups. The children with CP were found with significantly lower mean values of FFM and other components (all values *p* < 0.01). Likewise, BCMI and PA values were significantly lower in the study group of the children with CP (*p* < 0.05). No significant differences were found in FM (Table 2).

In the gender-related analysis, both boys and girls with CP showed significantly lower mean values of FFM, MM, BCM, and TBW (*p* < 0.01), compared to the controls. The BCMI and PA were significantly lower in the boys with CP (*p* < 0.01). In girls, although the differences in these parameters were similar, statistical significance was not reached. No significant differences were found in FM (Table 3).

**Figure 1:**
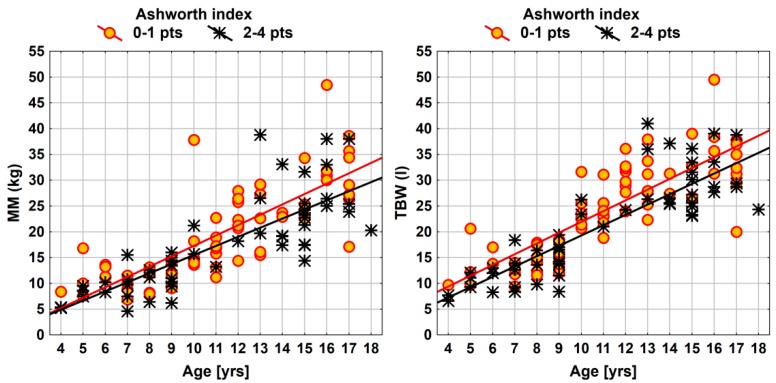
Effects of muscle tone in muscle mass and total body water in the children with CP. Abbreviations: MM—muscle mass; effect of muscle tone in body composition of children with CP shown using linear regression.

Table 4 shows relationships between muscle tone and measures of body composition in the children with CP. The children with higher scores in Ashworth scale were found with significantly lower values corresponding to selected body mass components (FM, FFM, MM, BCM, and TBW) (*p* < 0.05). The effects of muscle tone in body composition of children with CP are also presented using linear regression (for the parameters of MM and TBW) (Figure 1). The findings showed no significant correlations between the scores in Ashworth scale and the values of BCMI and PA.

**Figure 2:**
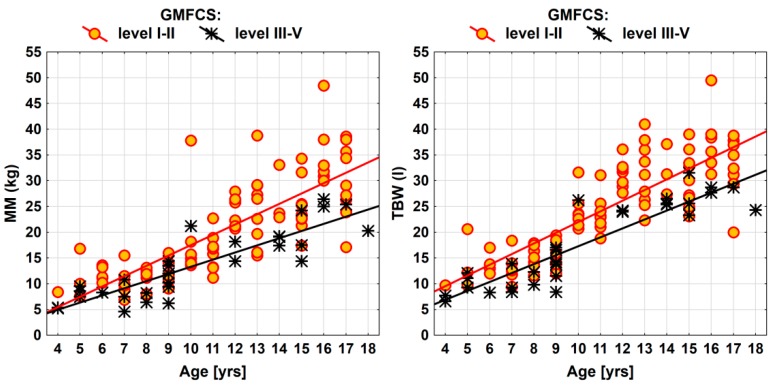
Effects of motor function in muscle mass and total body water in the children with CP.

Table 5 shows relationships between motor function and measures of body composition in the children with CP. The children with higher level in GMFCS were found with significantly lower values corresponding to selected body mass components (FFM, MM, BCM, BCMI, and TBW) (*p* < 0.05). The effects of motor function in the body composition of children with CP are also presented (for the parameters of MM and TBW) (Figure 2).

## 4. Discussion

This study involving 118 children with CP and healthy controls matched for age and gender, showed that the children with CP had significantly lower body composition parameters, also relative to gender. The children with increased muscle tone presented significantly lower values of specific components of body composition. To the best of our knowledge, supported by a review of international literature, our study assessing body weight composition in children with CP depending on the level of muscle tone measured with the Ashworth scale is the first such study. As one of the first, we also separately compare groups of boys and girls with CP to healthy controls. This is justified in connection with the significantly lower body mass indexes found in boys than in girls with CP. 

The study group of children was characterised by significantly lower values of body weight, height, and BMR (*p* < 0.05). These differences were observed in both girls and boys. In a study involving 69 Spanish children with CP representing a similar age group (10.46 y ± 0.43) and type of the condition (spastic), the findings showed significantly lower values of body weight in the group with the lowest level of functional capacities according to GMFCS, which correlated with lower values of FFM [34]. Lower values of these parameters and a potentially higher risk of undernutrition in children with CP were also reported in other parts of the world [35,36,37]. These findings are consistent with the related evidence published earlier. Children with CP achieve poorer results in centile grids [38] as they present lower baseline body weight, lower fat reserves [39], as well as lower resting energy expenditure (REE), compared to their healthy peers [40,41]. Altered energy requirements, in comparison to healthy children, result not only from lower activity and lower consumption of nutrients, but also from different muscle tone and body composition [42].

On the other hand, insufficient physical activity in children with CP may be associated with increased risk of body fat accumulation, and consequently various complications [43,44]. In the present case-control study, it was shown that selected body composition parameters (FFM, MM, BCM and TBW) and nutritional indicators (BCMI and PA) in children with CP were significantly different than in the healthy controls. The results of our study showed that the children with CP had significantly lower mean values of FFM, MM and BCM, expressed in kg (*p* < 0.001 respectively) and TBW expressed in L (*p* < 0.001). Moreover, our results showed significantly lower mean values of BCMI and PA (*p* < 0.05). Both boys and girls with CP had significantly lower mean FFM, MM, BCM, and TBW (*p* < 0.01) compared to control boys and girls. Significantly lower BCMI and PA parameters were found in boys with CP (*p* < 0.01) than in healthy boys. These results provide evidence that children with CP have lower energy reserves reflected by lower body composition parameters, which may result in a potentially higher risk of undernutrition and faster dynamics of changes in the body component due to existing malnutrition. No significant differences were observed in the measure of FM, also relative to the children’s gender. A study by Whitney et al., carried out in a group of 42 children with CP, demonstrated a lack of significant differences in FM (*p* = 0.10), with significantly lower values of FFM and FFMI (*p* < 0.05) [45]. An earlier cross-sectional study by Whithey et al., involving 18 children with mild spastic CP, aged 4–12 years, showed no group differences in FM and FFM (*p* < 0.05), however the findings also showed elevated central adiposity, especially in the visceral region. Based on the findings reported in 2018 by German researchers who analysed selected indicators of nutritional status, it was concluded that excess body fat in children with CP is less common than previously reported [46]. To the authors best knowledge, no analyses are available comparing the body composition of children with CP broken down by gender compared to healthy boys and girls. Oftedal et al. indicated a lower Body Fat% content in boys with CP compared to girls with CP (*p* < 0.001) [47].

Another interesting finding of the current study is that children with higher muscle tone, i.e., higher Ashworth index, presented significantly lower values of FM, FFM, MM, BCM and TBW (*p* < 0.05). The above effect of muscle tone in body composition of the children with CP is also visualised in Figure 2, by means of linear regression (for the measures of MM and TBW). These findings are linked with the increased caloric intake needs proportional to the increased muscle tone. This results in a reduction of all the components of body composition, including MM and TBW, which has been shown in our observations. In a study by Liu et al., baclofen was administered by the intrathecal route to 12 children with CP which resulted in decreased muscle tone (according to Modified Ashworth Scale) and reduced caloric intake needs, and consequently a lower risk of undernutrition [17]. A study by Tomoum et al. assessed nutritional status in children with CP and reported a statistically significant reduction in TBW, FM, FFM, fat percentage and BMR in the patients’ group compared to the control group (*p* < 0.01); the effect was particularly visible in the children with severe motor handicap and or motor dysfunction [12]. It may be difficult to interpret the evidence reported by the scarce related studies, including the current findings, because of the relatively lower combined values of FFM (MM and TBW) frequently result from lower MM [48] and bone mass [49] as a result of their immobility [18]. The present findings also show no interaction between muscle tone and gender, at a significance level of *p* < 0.05 in all the relevant measures of body composition. The current findings should be supported with further research to be conducted in a larger group of children with CP.

The results of our study also showed statistically significant relationships between GMFCS level and the values of body composition. Children with CP with levels III, IV and V GMFCS had significantly lower rates of FFM, MM, BCM, BCMI and TBW. Sung et al. also obtained similar results [50]. Oeffinger et al. mentioned that BIA for people with a higher level of GMFCS is very accurate and can be used to assess and monitor body composition in people with CP [51]. In a systematic review, Snik et al. emphasize that BIA is an important, simple and inexpensive method of assessing body mass composition, and studies of further validation of this method should be continued in a group of children with CP [39].

The strengths of the present study include the large sample size and the fact that we compared cases and controls relative to various determinants potentially contributing to the nutritional status. Our study is obviously not free from some limitations. Despite our best efforts and inclusion to our study as many participants as it was possible, reliability of bioimpedance measured by BIA may to a degree be questionable due to errors resulting from the fact that it may be difficult for the subjects to assume an identical position and to fully cooperate during the examination. Given the fact that important markers of nutritional status (albumin and/or pre-albumin level) were not assessed in all the children, we were not able to analyse the association of these variables with body composition components. Another limitation of the study is linked with the complex characteristics of the group (various types of the disorder), and this will also be taken into account in the future study involving a larger group of children.

## 5. Conclusions

Our findings showed lower parameters of body composition in children with CP compared to healthy children, and a decrease in the parameters coinciding with higher muscle tone in the study group. This observation suggests that it is necessary to measure and cure muscle tone while assessing the nutritional status of children with CP. We observed significantly lower body mass composition parameters in the group of boys with CP compared to healthy controls than in the groups of girls with CP and healthy girls. Based on our findings, we believe that there is a need for further study explaining the influence of muscle tone on nutrition and body composition separately for boys and girls with CP.

## Figures and Tables

**Table 1:** Anthropometric parameters of the study and control groups—estimation of difference between CP and controls using ANCOVA model with age as covariate.

Parameter	CP vs. Controls					
	Boys			Girls		
	Effect Size (95% CI)	*F*(1, 149)	*p* Value	Effect Size (95% CI)	*F*(1, 81)	*p* Value
Weight (kg)	−6.6 (−9.9; −3.2)	16.25	0.0002 ***	−5.8 (−9.7; −1.8)	7.99	0.0059 **
Height (cm)	−5.8 (−8.7; −3.0)	14.87	0.0001 ***	−7.0 (−10.3; −3.7)	17.32	0.0001 ***
BMI (kg/m^2^)	−1.6 (−2.6; −0.6)	10.16	0.0017 **	−0.8 (−2.4; 0.7)	1.08	0.3022
R-resistance	83.7 (54.7; 112.7)	31.99	<0.0001 ***	41.4 (−2.0; 84.7)	3.49	0.0652
Xc-reactance	4.3 (1.0; 7.6)	6.44	0.0122 *	0.4 (−3.1; 4.0)	0.06	0.8075
BMR	−452 (−621; −283)	27.45	<0.0001 ***	−306 (−494; −118)	10.21	0.0020 **

**Abbreviations:** BMI—body mass index; BMR-basal metabolic rate; * Bold characters indicate significant values (*p* < 0.05), ** *p* < 0.01, *** *p* < 0.001.

**Table 2:** Comparison of BIA results in study and control groups—estimation of difference between CP and controls using ANCOVA model with age as covariate.

Parameter	CP vs. Controls		
	Effect Size (95% CI)	*F*(1, 233)	*p* Value
FM (kg)	−0.9 (−2.4; 0.5)	1.57	0.2109
FMpct (%)	−0.4 (−2.9; 2.0)	0.12	0.7314
FFM (kg)	−5.3 (−7.0; −3.5)	34.87	<0.0001 ***
FFMpct (%)	0.3 (−2.1; 2.7)	0.06	0.8135
MM (kg)	−4.0 (−5.4; −2.6)	32.49	<0.0001 ***
MMpct (%)	−1.4 (−3.3; 0.5)	1.99	0.1602
BCM (kg)	−3.3 (−4.4; −2.1)	31.58	<0.0001 ***
BCMpct (%)	−1.9 (−3.1; −0.8)	10.45	0.0014 **
BCMI (kg/m^2^)	−0.8 (−1.2; −0.5)	20.24	<0.0001 ***
TBW (l)	−3.6 (−4.9; −2.3)	30.70	<0.0001 ***
TBWpct (%)	1.2 (−1.2; 3.6)	0.93	0.3366
PA (°)	−0.3 (−0.5; −0.1)	6.35	0.0124 *

**Abbreviations:** FM—fat mass; FFM—fat free mass; MM—muscle mass; BCM—body cell mass; BCMI—body cell mass index; TBW—total body water; PA—phase angle; * Bold characters indicate significant values (*p* < 0.05).

**Table 3:** Comparison of BIA results in study and control groups according to gender - estimation of difference between CP and controls using ANCOVA model with age as covariate.

Parameter	CP vs. Controls					
	Boys			Girls		
	Effect Size (95% CI)	*F*(1, 149)	*p* Value	Effect Size (95% CI)	*F*(1, 81)	*p* Value
FM (kg)	−0.5 (−2.3; 1.3)	0.35	0.5542	−1.7 (−4.1; 0.8)	1.74	0.1908
FMpct (%)	−0.3 (−3.4; 2.8)	0.04	0.8427	−0.6 (−4.3; 3.0)	0.12	0.7330
FFM (kg)	−5.9 (−8.1; −3.7)	27.22	<0.0001 ***	−4.1 (−6.2; −2.0)	14.18	0.0003 ***
FFMpct (%)	0.1 (−2.9; 3.1)	0.00	0.9486	0.6 (−3.0; 4.3)	0.12	0.7330
MM (kg)	−4.5 (−6.2; −2.8)	27.85	<0.0001 ***	−3.0 (−4.9; −1.2)	10.55	0.0017 **
MMpct (%)	−1.8 (−4.1; 0.5)	2.24	0.1364	−0.7 (−3.9; 2.4)	0.21	0.6502
BCM (kg)	−3.7 (−5.1; −2.3)	26.94	<0.0001 ***	−2.5 (−4.1; −1.0)	10.27	0.0019 **
BCMpct (%)	−2.1 (−3.5; −0.7)	8.29	0.0046 **	−1.6 (−3.5; 0.4)	2.55	0.1145
BCMI (kg/m^2^)	−1.0 (−1.5; −0.6)	24.95	<0.0001 ***	−0.4 (−1.1; 0.2)	1.69	0.1976
TBW (l)	−4.2 (−5.8; −2.6)	27.00	<0.0001 ***	−2.6 (−4.0; −1.2)	12.69	0.0006 ***
TBWpct (%)	1.1 (−1.9; 4.1)	0.48	0.4900	1.4 (−2.1; 4.8)	0.62	0.4345
PA (°)	−0.3 (−0.6; −0.1)	6.96	0.0092 **	−0.2 (−0.6; 0.2)	0.79	0.3752

**Abbreviations:** FM—fat mass; FFM—fat free mass; MM—muscle mass; BCM—body cell mass; BCMI—body cell mass index; TBW—total body water; PA—phase angle. * Bold characters indicate significant values (*p* < 0.05).

**Table 4:** Selected components of body composition in the children with CP relative to muscle tone level according to Ashworth scale.

Parameter	Ashworth Index (2–4 vs. 0–1 pts)		
	Effect Size (95% CI)	*F*(1, 115)	*p* Value
FM (kg)	−2.7 (−4.8; −0.5)	5.88	0.0168 *
FMpct (%)	−0.9 (−4.7; 3.0)	0.18	0.6680
FFM (kg)	−3.3 (−5.8; −0.9)	6.96	0.0095 **
FFMpct (%)	1.1 (−2.8; 4.9)	0.31	0.5793
MM (kg)	−2.1 (−4.1; 0.0)	3.97	0.0488 *
MMpct (%)	0.8 (−2.4; 4.0)	0.25	0.6178
BCM (kg)	−1.7 (−3.4; 0.0)	3.97	0.0486 *
BCMpct (%)	−0.1 (−2.2; 1.9)	0.01	0.9046
BCMI (kg/m^2^)	−0.4 (−1.0; 0.2)	1.63	0.2049
TBW (l)	−2.7 (−4.6; −0.9)	8.34	0.0046 **
TBWpct (%)	0.1 (−3.8; 3.9)	0.00	0.9790
PA (°)	0.0 (−0.4; 0.4)	0.04	0.8477

**Abbreviations:** FM—fat mass; FFM—fat free mass; MM—muscle mass; BCM—body cell mass; BCMI—body cell mass index; TBW—total body water; PA—phase angle; * Bold characters indicate significant values (*p* < 0.05); the significance of the inter-group differences was assessed using analysis of covariance which took into account age as an accompanying variable. Analysis is based on a dichotomous distinction of two groups, according the score in Ashworth Scale (one group with scores of 0–1; the other group with scores of 2–4).

**Table 5:** Selected components of body composition in the children with CP relative to motor function level according to GMFCS.

Parameter	GMFCS (level III-V vs. I-II)		
	Effect Size (95% CI)	*F*(1, 115)	*p* Value
FM (kg)	−1.2 (−3.8; 1.3)	0.90	0.3453
FMpct (%)	2.7 (−1.8; 7.1)	1.38	0.2430
FFM (kg)	−6.2 (−8.9; −3.5)	20.26	<0.0001 ***
FFMpct (%)	−2.5 (−6.8; 1.9)	1.20	0.2749
MM (kg)	−4.6 (−6.8; −2.3)	16.27	0.0001 ***
MMpct (%)	−3.3 (−6.9; 0.3)	3.14	0.0790
BCM (kg)	−3.8 (−5.6; −1.9)	16.05	0.0001 ***
BCMpct (%)	−2.4 (−4.7; 0.0)	3.92	0.0501
BCMI (kg)	−0.9 (−1.5; −0.2)	7.01	0.0092 **
TBW (l)	−4.9 (−6.9; −2.9)	22.77	<0.0001 ***
TBWpct (%)	−2.6 (−7.0; 1.8)	1.39	0.2407
PA (kg)	−0.4 (−0.9; 0.0)	3.12	0.0801

**Abbreviations:** FM—fat mass; FFM—fat free mass; MM—muscle mass; BCM—body cell mass; BCMI—body cell mass index; TBW—total body water; PA—phase angle. * Bold characters indicate significant values (*p* < 0.05); the significance of the inter-group differences was assessed using ANCOVA model with GMFCS as a group variable and age as a control variable.

## Supplementary Materials

The following are available online at https://www.mdpi.com/2072-6643/12/3/864/s1, Table S1: Sociodemographic, anthropometric and clinical factors of the study and control groups.
