# Supplementary material for: The Relationship between Body Composition and Muscle Tone in Children with Cerebral Palsy: A Case-Control Study
Source: Nutrients. 2020 Mar 24;12(3):864. doi: 10.3390/nu12030864 (PMC7146599; doi:10.3390/nu12030864)
Supplement: Supplementary file 1 [file nutrients-12-00864-s001.pdf]

Supplementary Materials

Table S1. Sociodemographic, anthropometric and clinical factors of the study and control groups.

**Parameter**	**CP**	**Controls**	***p value***
	**Mean (DS)** *n* **(%)**		
**Sex**			
Boys	76 (64.41)	76 (64.41)	$1.000^{a)}$
Girls	42 (35.59)	42 (35.59)	
**Aged**	11.10 (3.83)	11.10 (3.83)	$1.000^{b)}$
**Place of residence**			
Urban area	75 (63.56)	61 (51.69)	$0.065^{a)}$
Rural area	43 (36.44)	57 (48.31)	
**Weight (kg)**	38.50 (17.45)	44.77 (18.18)	$0.007^{b)}$
**Height (cm)**	143.37 (22.04)	149.63 (20.39)	$0.040^{b)}$
**BMI (kg/m^2)**	17.69 (4.06)	19.03 (3.73)	$0.003^{b)}$
**R-resistance**	719.67 (124.57)	651.14 (107.17)	$< 0.001^{b)}$
**Xc-reactance**	67.13 (11.66)	64.27 (7.60)	$0.080^{b)}$
**BMR**	4 966.73 (919.54)	5 366.94 (1 075.47)	$0.004^{b)}$
**Type of the cerebral palsy**			
Spastic	94 (79.7)	-	-
Mixed	16 (13.)6	-	-
Ataxic	6 (5.1)	-	-
Unclassified	2 (1.7)	-	-
**Way of feeding**			
Oral	115 (97.5)	-	-
Enteral feeding by Percutaneous Endoscopic Gastrostomy	3 (2.5)	-	-
**Ashworth Scale**			
Level 0	13 (11.0)	-	-
Level 1	54 (45.8)	-	-
Level 2	35 (29.7)	-	-
Level 3	14 (11.9)	-	-
Level 4	2 (1.7)	-	-
**GMFCS**			
Level 1	32 (27.1)	-	-
Level 2	56 (47.5)	-	-
Level 3	4 (3.4)	-	-
Level 4	17 (14.4)	-	-
Level 5	9 (7.6)	-	-

a) – Chi-square test. b) – Mann – Whitney test.
